# Medical research productivity in the Arab countries: 2007-2016 bibliometric analysis

**DOI:** 10.7189/jogh.08.020411

**Published:** 2018-12

**Authors:** Rola El Rassi, Lokman I Meho, Acile Nahlawi, Johnny S Salameh, Ali Bazarbachi, Elie A Akl

**Affiliations:** 1 Clinical Research Institute, American University of Beirut Medical Center, Beirut, Lebanon; 2 University Libraries, American University of Beirut, Lebanon; 3 American University of Beirut, Lebanon; 4 Department of Neurology, American University of Beirut Medical Center, Beirut, Lebanon; 5 Department of Internal Medicine, American University of Beirut Medical Center, Beirut, Lebanon; 6 Department of Anatomy, Cell Biology and Physiological Sciences, American University of Beirut Medical Center, Beirut, Lebanon

## Abstract

**Background:**

The aim of this study was to assess recent trends in medical research productivity in Arab countries.

**Methods:**

We collected bibliometric data for the world countries, Arab countries, and Arab institutions for 2007-2016, using Essential Science Indicators, Journal Citation Reports, and Web of Science database. We collected the number of published papers overall and per year, citations per paper, and number of papers published in top quartile and top 10% journals. For the 10 most productive institutions, we additionally collected the number of papers with correspondence authors affiliated with the institution.

**Results:**

The Arab world produced 189 papers per one million people, about a quarter of the value for other world countries. Four Arab countries (Qatar, Tunisia, Lebanon, and Kuwait) produced more than 695 papers per one million people, exceeding the world average. The average number of citations per paper was 9.2; it rose to more than 15 for papers with international collaboration. At the institutional level, the number of citations showed upward trends, with six institutions having an average citation per paper higher than that of all Arab countries. For the 10 most productive institutions in Arab countries, the percentage of papers involving international collaborations ranged from 42% to 79%; of these, 9% to 29% were led by authors from the same institution. For these 10 most productive institutions, the percentage of papers published in the top quartile journals and with a lead/corresponding author from the institution ranged from 7 to 32%; that percentage drops to 1% to 10% for papers published in top 10% journals.

**Conclusions:**

Although medical research output in Arab countries at both the country and the institution levels has increased over the past 10 years, it is still lagging behind the rest of the world. The percentage of papers involving international collaborations was relatively high, but the majority of these papers were led by authors from outside the local institution, particularly when published in the top 10% journals.

Research in the medical sciences plays an important role in a country’s economic growth, long-term sustainable development, and improvement in the standards of living and quality of life [1]. Governments across Europe as well as the United States have heeded these claims and have increased research spending in an effort to aide their ailing economies [1].

The situation is different in Arab countries however. Arab countries are lagging behind in the number of original research publications, number of publications in top journals and citation frequency [2], to name a few. The number of medical publications from Arab institutions in all 22 Arab countries between 1996 and 2012 was 76 417 reports, which is equivalent to almost half that of Turkey and equal to only 4% of medical publications from United States based institutions [3]. This lag holds true across a number of other publications that have also assessed medical research in Arab countries and found it to be lagging [2,4-6].

It is therefore essential to quantify current research output as it constitutes the basis for strategic planning and decisions making [7]. Bibliometric analysis, now a widely accepted method for assessing research in many fields, is employed to quantify and assess research output and also depict its growth and spread [8]. Comparisons of bibliometric characteristics between countries and institutions can reveal differences in research orientations, capacities, and collaboration patterns [9]. Additionally, universities and academic institutions increasingly rely on scientific analyses for making decisions regarding hiring, promotion, tenure, funding, and salary increases [9-13].

Published studies assessing medical research in Arab countries have mainly used PubMed to quantify productivity [2,4-6]. Since improving research productivity in Arab countries necessitates better understanding of its current status, we aim to assess more recent trends in medical research productivity in Arab countries, in terms of both quantity and quality using Essential Science Indicators, Journal Citation Reports, and Web of Science database.

## METHODS

### Overall design

This study compared medical research output: 1) between Arab countries combined and the rest of the world; 2) across individual Arab countries; and 3) across institutions in the region (hereafter referred to as “institutions”).

### Eligibility criteria

We used the following eligibility criteria:

- Countries: we included all 22 countries classified as Arab countries according to the Arab League [14];
- Institutions: we included the 10 most productive universities or research hospitals in Arab countries. We could have chosen a larger pool, for example, 15, 20, or 25, but that would have included institutions with less than 100 papers per year from 2007 to 2016, a number that might be too low to make accurate citation-based assessments;
- Research subject fields: we used data from the 8 broad medical fields as defined in Clarivate Analytics’ *Essential Science Indicators* database, including: biology & biochemistry, clinical medicine, immunology, microbiology, molecular biology & genetics, neuroscience & behavior, pharmacology & toxicology, and psychiatry/psychology;
- Time period: we focused on the January 2007 – December 2016 time period to include enough data to make the findings reliable while reflecting the recent state of medical research in Arab countries;
- Types of documents: we included only ‘article’ and ‘review' document types as classified by the Web of Science database. We excluded letters, editorials, abstracts and other types of documents. Of note, the Web of Science classifies ‘case reports’ as articles.

### Source of data

We collected data in November 2017 using Essential Science Indicators, Journal Citation Reports, and Web of Science database. To identify all of eligible journals in the database, we used the 2007-2016 editions of *Journal Citation Reports* and included only those journals that were classified by *Essential Science Indicators* under the aforementioned eight broad subject categories. We included a total of 4530 journals (of which 421 ceased publication or changed names at some point during the period 2007-2016).

### Data collected

We collected for each country the following data:

- Population size - using World Bank DataBank [15];
- Number of published papers per year;
- Number of papers with international collaboration for Arab countries; that is, papers that include at least two authors with respective affiliations from two different countries. We sub-categorized collaborations according to whether they are with: 1) Arab countries; 2) the European Union (including the United Kingdom); and 3) Canada and the United States;
- Citations per paper.

We also identified the top 25 medical research journals according to the number of papers published in those journals with at least one author affiliated with an institution from Arab countries, and specified their most recent impact factor scores.

We collected for the 10 most productive institutions in Arab countries the following data:

- Number of published papers per year;
- Citations per paper;
- Number of published papers involving international collaboration;
- Number of papers published in top quartile (Q1) and top 10% journals in their respective fields, as classified by the *Journal Citation Reports.* These were identified as follows: We first identified the top 25% and top 10% journals in each of *Journal Citation Reports’* 200 plus subject areas and then matched the titles against the 4530 journals classified as medical research by Essential Science Indicators to come up with a list of top 25% and top 10% journals in medicine;
- Number of papers with correspondence authors affiliated with institutions in the Arab states.

### Data analysis

For all analyses, we used the number of eligible publications over a 10-year period (2007-2016). The first set of analyses compared the research output of Arab countries as a whole to the world. We calculated for each country and for the Arab countries combined:

- World share: the percentage of papers published by the country out of the total number of papers published worldwide. For Arab countries, we calculated Arab world share which is the percentage of papers published by the country out of the total number of papers published by all Arab country researchers;
- Papers per 1 million people: which is equivalent to the number of papers published per country multiplied by 1 000 000-population and divided by the population of that country;
- Percentage of world population: the percentage population of a country as compared to the world;
- Total number of papers published during 2007-2016 and per year for Arab countries combined and other world countries;

For Arab countries alone, we calculated:

- The percentage of papers produced through international collaborations with other Arab countries, countries from the European Union, and North America for each country and for the Arab countries combined;
- Citation rate stratified by whether paper involved an international collaboration or not.

The second set of analyses compared the research output of institutions in Arab countries. We calculated for each institution:

- Total number of papers published during 2007-2016 for 10 most productive Arab institutions;
- Percent national contribution: the percentage of papers published by the institution out of the total number of papers published in the respective country;
- Percentage of papers published in top quartile (Q1) and top 10% journals in their respective fields;
- Percentage of published papers involving international collaborations;
- Percentage of papers with correspondence authors affiliated with these institutions; and then stratified by percentage of papers published in top quartile (Q1) and top 10% journals.

We did not explore the distribution of our data due to its huge size and the limitations in extraction; extracted as a lump rather than separate variables. We therefore calculated averages where applicable.

## RESULTS

### Arab counties vs world

We identified a total of 76 978 papers published by research groups in Arab countries between 2007 and 2016. **Table 1** shows the medical research output for the top 25 world countries, ranked in descending order by papers per million people, and for all Arab countries combined. The papers published in Arab countries as a whole constituted 1.6% of the world output. As for the number of papers per million people, research groups in Arab countries published 189 papers per one million people as compared to 695 papers per one million by researchers in all world countries, excluding Arab countries.

**Table 1:** Medical research output by world countries; 2007-2016; countries ranked in descending order by papers per 1 million people and limited to first 25 countries

Rank	Countries	Papers per country*	World share	Population†	% of world population	Papers per 1 million	Citations per paper*
1	Switzerland	106 969	2.2%	8 372 098	0.11%	12 777	23.7
2	Denmark	65 119	1.3%	5 731 118	0.08%	11 362	22.4
3	Netherlands	170 420	3.5%	17 018 408	0.23%	10 014	22.8
4	Sweden	98 873	2.0%	9 903 122	0.13%	9984	21.6
5	Australia	189 549	3.8%	24 127 159	0.32%	7856	18.4
6	Belgium	79 630	1.6%	11 348 159	0.15%	7017	22.9
7	Canada	247 172	5.0%	36 286 425	0.49%	6812	20.7
8	United Kingdom	424 060	8.6%	65 637 239	0.88%	6461	22.7
9	Israel	54 758	1.1%	8 547 100	0.11%	6407	18.8
10	Austria	54 654	1.1%	8 747 358	0.12%	6248	20.3
11	United States	1 659 565	33.7%	323 127 513	4.34%	5136	21.3
12	Germany	383 518	7.8%	82 667 685	1.11%	4639	19.3
13	Italy	246 987	5.0%	60 600 590	0.81%	4076	18.4
14	Greece	42 761	0.9%	10 746 740	0.14%	3979	16.2
15	France	236 511	4.8%	66 896 109	0.90%	3535	20.0
16	Spain	162 440	3.3%	46 443 959	0.62%	3498	17.1
17	Taiwan	75 670	1.5%	23 500 000	0.32%	3220	11.6
18	South Korea	157 790	3.2%	51 245 707	0.69%	3079	11.0
19	Japan	313 600	6.4%	126 994 511	1.71%	2469	13.9
20	Poland	63 534	1.3%	37 948 016	0.51%	1674	11.7
21	Turkey	106 279	2.2%	79 512 426	1.07%	1337	6.4
22	Brazil	135 175	2.7%	207 652 865	2.79%	651	10.0
23	Iran	50 479	1.0%	80 277 428	1.08%	629	6.6
24	China	448 360	9.1%	1 378 665 000	18.53%	325	9.4
	**Arab countries**	**76 978**	***1.6%***	**406 452 690**	***5.46%***	***189***	***9.2***
25	India	126 029	2.6%	1 324 171 354	17.79%	95	9.3
	**WORLD (excluding Arab countries)**	**4** **892** **030**		**7** **035** **682** **888**	**94.54%**	**695**	**15.1**

*Source: Essential Science Indicators (October 1, 2017 edition), covering papers and citations for the period January 1, 2007 – August 31, 2017.

†Source: World Bank DataBank.

**Figure 1:**
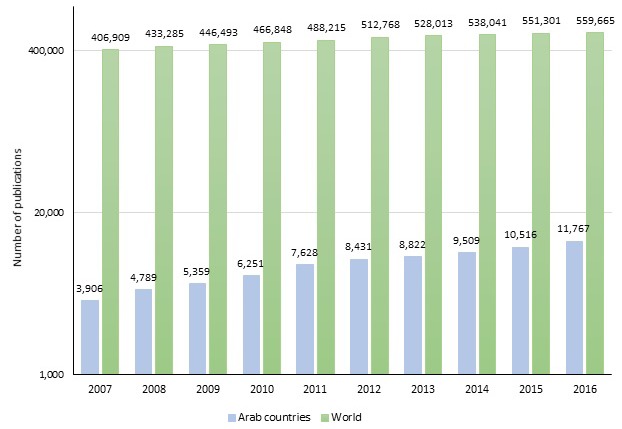
Number of publications in medical research in the Arab countries as a whole vs the world per year between 2007and 2016.

**Figure 1** further depicts the trend of medical research productivity in number of papers per year in Arab countries combined and in the world. The productivity of researchers in Arab countries has increased 3-fold between 2007 and 2016.

### Arab countries amongst each other

**Table 2** shows the medical research output of researchers in individual Arab countries. Research groups from Qatar, Tunisia, Lebanon, and Kuwait (in descending order) had a total number of papers per one million people above the world average of 695 papers per million. The number of citations per paper was higher than the world average of 15.1 in Sudan alone. Arab countries that had publications with a citation rate higher than that of all Arab countries combined were in descending order Sudan, Lebanon, United Arab Emirates, Qatar, Oman, Saudi Arabia, Jordan and Iraq.

**Table 2:** Medical research output of all Arab countries 2007-2016; countries ranked in descending order by papers per 1 million people

World Rank	Countries	Papers per country*	Arab world share	Papers per 1 million	Citations per paper*
69	Qatar	2971	3.9%	1156	11.4
50	Tunisia	8769	11.4%	769	8.4
54	Lebanon	4504	5.9%	750	13.4
71	Kuwait	2862	3.7%	706	8.7
39	Saudi Arabia	21 897	28.4%	678	9.6
108	Bahrain	682	0.9%	479	NR†
58	United Arab Emirates	3927	5.1%	424	12.1
62	Jordan	3592	4.7%	380	9.5
85	Oman	1527	2.0%	345	11.1
36	Egypt	24 692	32.1%	258	7.8
113	Palestine	613	0.8%	128	NR†
56	Morocco	4027	5.2%	114	7.3
119	Libya	561	0.7%	89	NR†
80	Algeria	1902	2.5%	47	9.1
106	Syria	710	0.9%	39	NR†
186	Djibouti	36	0.0%	38	NR†
90	Iraq	1279	1.7%	34	9.3
89	Sudan	1280	1.7%	32	16.7
198	Comoros	19	0.0%	24	NR†
120	Yemen	529	0.7%	19	NR†
178	Mauritania	58	0.1%	13	NR†
192	Somalia	24	0.0%	2	NR†
**TOTAL**	**ARAB COUNTRIES**	**76** **978**	**100.0%**	**189**	**9.2**

NR – not reported.

*Source: Essential Science Indicators (October 1, 2017 edition), covering papers and citations for the period January 1, 2007 – August 31, 2017.

†Not reported because the number of papers published by the country was too small to accurately calculate its citation rate.

Fifty one percent of papers published by research groups in Arab countries did not involve any international collaborations (**Table 3**) and had an average of 5.4 citations per paper. Twenty seven percent of papers involved collaborations with the European Union and 19% with Canada and United States, with an average citation rate of 14.8 per paper and 18.2 per paper, respectively. Researchers in Qatar, United Arab Emirates, Saudi Arabia, Lebanon, Jordan and Egypt – in descending order – collaborated with Europe and Canada and the United States on more than 50% of their publications. Researchers in Arab countries mostly submitted their publications to journals of low impact; only four of those journals ranked in the top quartile and only five have an impact factor above 3.0 (**Table 4**). Also, only 24% of Arab countries’ papers are published in top quartile journals in comparison to 38% for the rest of the world.

**Table 3:** Collaborations by individual Arab countries; 2007-2016; countries ranked in descending order by total research output*

	Total research output		Papers without international collaboration		Papers with international collaboration with							
**other Arab countries**		**European Union**		**Canada and United States**		**Overall**	
**Country †**	**Papers per country**	**Citations per paper†**	**%**	**Citations per paper†**	**%**	**Citations per paper†**	**%**	**Citations per paper†**	**%**	**Citations per paper†**	**%**	Citations per paper*
Egypt	24 692	8.3	49	6.0	21	8.1	18	16.2	16	16.9	51	10.8
Saudi Arabia	21 897	9.7	32	5.4	25	8.5	21	18.5	23	21.2	68	12.0
Tunisia	8769	8.3	55	5.1	9	18.2	37	14.1	6	34.0	45	12.8
Lebanon	4504	15.0	36	8.1	15	26.0	34	26.4	33	27.4	64	19.3
Morocco	4027	7.7	59	3.1	9	30.9	33	16.2	8	36.0	41	14.8
United Arab Emirates	3927	13.2	23	7.2	21	15.6	36	21.0	30	24.1	77	15.2
Jordan	3592	11.2	46	6.1	18	22.4	21	27.1	23	26.9	54	16.0
Qatar	2971	12.5	15	7.6	22	20.4	47	18.2	36	20.8	85	13.6
**Arab world**	**76978**	**9.2**	**51**	**5.4**	**–**	**–**	**27**	**14.8**	**19**	**18.2**	**49**	**13.0**

*This table is limited to the 8 most published countries with most research output. The papers by other countries are too few to accurately examine the impact of their international collaborations.

†Source: Web of Science (November 2017), covering papers for the period 2007-2016 cited from January 2007 up to November 2017.

**Table 4:** List of the top 25 medical research journals in which researchers from Arab countries publish, ranked in descending order by number of papers published with at least one author affiliated with an institution from the Arab countries

Rank	Name of journal	Number of papers	Quartile	IF*
1	Saudi Medical Journal	1412	4	0.709
2	Life Science Journal - Acta Zhengzhou University Overseas Edition	903	4	0.165
3	Annals of Saudi Medicine	580	4	0.558
4	African Journal of Biotechnology	444	4	0.573
5	Parasitology Research	431	2	2.329
6	Biomed Research International	361	2	2.476
7	Medical Principles and Practice	332	2	1.469
8	Natural Product Research	328	3	1.828
9	African Journal of Microbiology Research	327	4	0.539
10	Medicinal Chemistry Research	312	4	1.277
11	International Journal of Biological Macromolecules	308	1	3.671
12	Kuwait Medical Journal	307	4	0.089
13	Neurosciences	302	4	0.552
14	Archives de Pediatrie	299	4	0.372
15	Saudi Journal of Biological Sciences	292	2	2.564
16	Asian Pacific Journal of Cancer Prevention	246	3	2.514
17	Natural Product Communications	243	4	0.773
18	International Journal of Pharmaceutics	236	1	3.061
19	Journal of Infection in Developing Countries	235	4	1.353
20	Cochrane Database of Systematic Reviews	227	1	6.264
21	Bioresource Technology	225	1	5.651
22	Pakistan Journal of Medical Sciences	224	3	0.696
23	Annales de Biologie Clinique	224	4	0.225
24	Saudi Pharmaceutical Journal	214	3	2.302
25	World Journal of Gastroenterology	208	2	3.365

IF – 2015 impact factor

### Institutions in Arab countries

**Figure 2:**
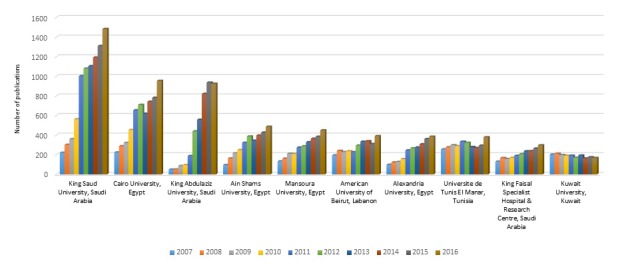
Number of publications in medical research across the years of the 10 most productive institutions in Arab countries.

The ten most productive institutions in the Arab countries are listed in **Table 5**. These institutions were from Egypt (n = 4), Saudi Arabia (n = 3), Lebanon (n = 1), Kuwait (n = 1), and Tunisia (n = 1). Research groups from the Kuwait University in Kuwait and the American University of Beirut in Lebanon had a greater than 50% share of research productivity contribution per country. Papers published by six of the 10 most productive institutions had an average citation per paper higher than that of Arab countries as a whole; none had an average citation per paper higher than world average. **Figure 2** depicts the productivity trend in medical research of researchers in these institutions. Almost all institutions showed upward trend, although to different degrees. Research productivity of groups at Kuwait University in Kuwait showed an almost stagnant trend.

**Table 5:** Percent national contribution and citations per paper of the 10 most productive institutions in Arab countries between 2007 and 2016; countries ranked in descending order of the number of citations per paper

Rank	Institution and country	Papers per institution	Papers per country	Percent national contribution	Citations per paper*
1	American University of Beirut – Lebanon	2794	4433	63	13.8
2	King Abdulaziz University – Saudi Arabia	4151	21897	19	13.0
3	King Faisal Specialist Hospital & Research Centre – Saudi Arabia	2057	21897	9	12.7
4	Kuwait University – Kuwait	1862	2862	65	10.4
5	Cairo University – Egypt	5750	24 692	23	9.5
6	Mansoura University – Egypt	2803	24 692	11	9.3
7	Ain Shams University – Egypt	3089	24 692	13	9.0
8	Alexandria University – Egypt	2345	24 692	9	8.8
9	King Saud University – Saudi Arabia	8623	21 897	39	8.3
10	Universite de Tunis El Manar – Tunisia	2998	8769	34	8.0

*Source: Web of Science (November 2017), covering papers for the period 2007 – 2016 cited from January 2007 up to November 2017.

The research output of four institutions ranked higher than the average of Arab countries as a whole in the percentage of papers published in top quartile journals (**Table 6**). More than 10% of papers produced by researchers at King Faisal Specialist Hospital & Research Centre in Saudi Arabia and the American University of Beirut in Lebanon were published in top 10% journals (**Table 6**).

**Table 6:** Percentage of papers in first quartile (Q1) and top 10% journals of the ten most productive institutions in Arab countries between 2007 and 2016; countries ranked in descending order by percentage of top 10% papers

Rank	Institution and country	Papers per institution	Percentage of papers in Q1 journals	Percentage of papers in top 10% journals
1	King Faisal Specialist Hospital & Research Centre – Saudi Arabia	2057	35	13.1
2	American University of Beirut – Lebanon	2794	36	12.7
3	King Abdulaziz University – Saudi Arabia	4151	29	9.6
4	Alexandria University – Egypt	2345	24	7.2
5	Cairo University – Egypt	5750	23	6.9
6	King Saud University – Saudi Arabia	8623	22	6.6
7	Mansoura University – Egypt	2803	21	6.4
8	Kuwait University – Kuwait	1862	20	5.9
9	Ain Shams University – Egypt	3089	22	5.7
10	Universite de Tunis El Manar – Tunisia	2998	13	4.4

**Figure 3:**
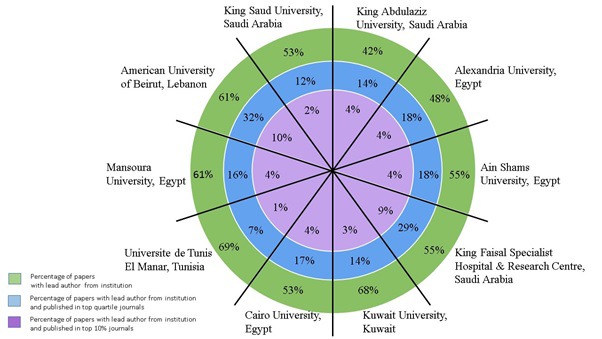
Percentages of papers with lead/correspondence author from institution (green band), published in first quartile journals (blue band), and published in the top 10% journals (purple band) for the 10 most productive institutions in Arab countries. Surface area is not proportional to percentage and institutions have not been ranked in any specific order.

The percentage of papers with lead/correspondence author from the same institution was higher than 50% in eight Arab institutions (**Figure 3**). Only two of these institutions’ researchers had more than 20% of their papers published in top quartile journals. Researchers from one institution only had more than 10% of their papers published in the top 10% journals.

**Figure 4:**
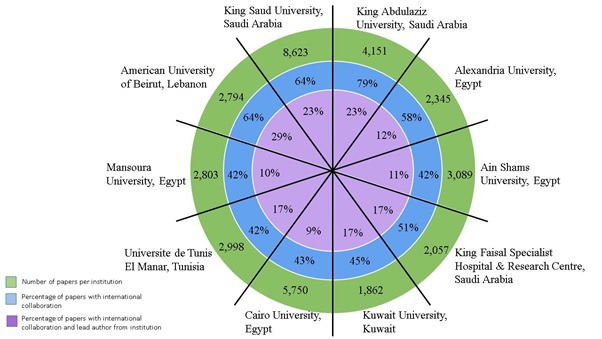
Papers with international collaboration for the 10 most productive institutions in Arab countries (green band), with percentage with international collaboration (blue band), and percentage with both international collaboration and lead authorship from the institution (purple band). Surface area is not proportional to percentage and institutions have not been ranked in any specific order. The percentage of papers with international collaboration and lead/correspondence author from institution was calculated from the total number of papers per institution.

**Figure 4** illustrates the international collaborations between research groups for the ten most productive institutions, which ranged from 42% to 79% across the 10 institutions. The percentage of papers involving international collaborations and led by authors from the same institution ranged between 9% and 29% across the 10 institutions.

## DISCUSSION

In summary, this study used bibliometric indicators to describe medical research productivity in 22 Arab countries during the last decade. Over the 2007-2016 time period, the number of medical research publications per one million people for researchers in Arab countries was almost about a fourth of that of the world. However, medical research productivity has been increasing in these countries at a faster rate, relative to the rest of the world during the same time period.

The study has a number of strengths. First, and to our knowledge, this is the first article to analyze the quality and quantity of medical research in Arab countries using bibliographic resources such as Essential Science Indicators, Journal Citation Reports, and Web of Science database. Previous studies on research productivity in Arab countries have mainly used the Medline database [2-6]. Nonetheless, our finding of paucity in medical research productivity is consistent with the findings of those studies [2-6]. Second, we adjusted the raw counts of publications by population number to avoid misleading results. As such, our study showed that productivity is higher in small countries, such as Qatar, Tunisia, Kuwait and Lebanon than in Saudi Arabia and Egypt. Other investigators reported similar findings for small countries when adjusting for population size [4,5].

The study also has a number of limitations. Although we included papers from a sizeable number of medical research journals (as classified by *Essential Science Indicators*), there is probably a number of local medical journals that were not captured by the used databases. However, we believe that including more journals would increase the numbers but in a proportional way that would not affect the interpretation of the results. Finally, the inclusion of “case reports” in this study (given the Web of Science classifies them as articles) could have lowered the number of citations per papers, particularly since major journals do not accept to publish case reports.

One of our major findings is the paucity in medical research productivity in Arab countries. Reasons stipulated to explain this paucity include political instability in these countries, regional conflicts [5], lack of proper research infrastructure and equipment [3], lack of freedom and democracy, brain drain, lack of funding, and difficulty of publishing in high impact journals [16,17].

At the institutional level, almost all institutions showed upward trends, although to different degrees, with six of them having average citations per paper higher than that of Arab countries as a whole. The ranking of these institutions changes when focusing on quality as opposed to quantity indicators.

It is noteworthy that papers involving international collaborations had a 3-fold higher citation rate than those not involving international collaborations. This is consistent with findings from a number of other studies conducted in other settings [18-20]. This finding highlights the importance of international collaboration, particularly when considering advantages such as increased visibility, and more opportunities for securing funding.

Researchers in Arab countries collaborate primarily with the United States and Canada and countries in the European Union, which could mostly be due to having received their education or training in said countries. The intriguing finding was that a relatively small percentage of papers involving collaboration and published in top journals, were led by authors from local institutions. This raises concerns about institutions in Arab countries offering affiliations to international investigators as a way to improve their medical research productivity metrics.

There is a need to maintain the momentum of increasing number of publications while improving their quality. Capacity building in conducting medical research would be essential for that purpose. Oliver et al. (2015) has proposed conducting capacity building at the levels of individuals, research teams, organizations, and country wide. Although that approach was conceived for systematic reviews, it does apply to the wider research field [21].

Additional national level policies to enhance medical research could include strengthening national funding programs and building the research infrastructure (including regulatory and supervisory bodies). While there are many reasons for institutions to pursue regional as well as international research collaborations, a possible increase in citation rate would be an extra advantage. Other institutional level interventions include instatement of tenure, intramural funding programs, establishing doctoral and postdoctoral programs, and faculty compensation schemes that incentivize research [12,13].

Institutions and authors can also use social media to disseminate their research output. The use of social media will benefit both the institution and the author by making the publications easier to find and access [22] and presumably increase citation impact [23]. A study by Thelwall et al (2013) evaluated the impact of specific altmetrics on citation rate and provided strong evidence that six of the 11 altmetrics assessed (tweets, Facebook wall posts, research highlights, blog mentions, mainstream media mentions and forum posts) associated with citation counts, at least in medical and biological sciences [24].

## CONCLUSIONS

Although the medical research output of Arab countries has increased over the past 10 years, it is still lagging behind the rest of the world. There is a need for systemic interventions at the country and institutional levels to maintain the momentum and improve the quality of the output.
